# Distribution and Risk Factors Associated With Tilapia Parvovirus (TiPV) Presence in Red Hybrid Tilapia (*Oreochromis* spp.) Farms in Thailand

**DOI:** 10.1155/tbed/6618755

**Published:** 2025-02-10

**Authors:** Benya Chatkaewchai, Win Surachetpong, Suporn Thongyuan, Methanan Kamklang, Sudtisa Laopiem, Sakuna Pattanakunanan, Visanu Boonyawiwat, Theeraporn Pulpipat

**Affiliations:** ^1^Master of Science Program in Veterinary Clinical Studies, Faculty of Veterinary Medicine, Kamphaeng Saen Campus, Nakhon Pathom 73140, Thailand; ^2^Department of Veterinary Microbiology and Immunology, Faculty of Veterinary Medicine, Kasetsart University, Bangkok 10900, Thailand; ^3^Laboratory of Biotechnology, Chulabhorn Research Institute, Bangkok 10210, Thailand; ^4^Department of Veterinary Public Health, Faculty of Veterinary Medicine, Kasetsart University, Kamphaeng Saen Campus, Nakhon Pathom 73140, Thailand; ^5^Kamphaeng Saen Veterinary Diagnostic Center, Faculty of Veterinary Medicine, Kasetsart University, Kamphaeng Saen Campus, Nakhon Pathom 73140, Thailand; ^6^Department of Farm Resources and Production Medicine, Faculty of Veterinary Medicine, Kasetsart University, Kamphaeng Saen Campus, Nakhon Pathom 73140, Thailand

**Keywords:** disease control, novel parvovirus, tilapia farming, unusual mortality

## Abstract

Emerging viral diseases, such as tilapia parvovirus (TiPV), are having a significant economic impact on global tilapia aquaculture. TiPV is responsible for the mass mortality of Nile tilapia (*Oreochromis niloticus*) and red hybrid tilapia (*Oreochromis* spp.) in China, India and Thailand. We, therefore, aimed to determine the current status of TiPV infection and distribution and the risk factors associated with TiPV infection in red hybrid tilapia farms in Thailand. In this cross-sectional study, a total of 101 samples, each comprising five moribund fish, were collected from 40 red hybrid tilapia farms across various provinces in Thailand between September 2022 and March 2024. The data on the farm characteristics and management practices were obtained via questionnaires and direct observation. A total of 23 factors were assessed, including six related to farm characteristics, 13 associated with farm management practices and four concerning the presence of other pathogens. The data from 101 samples were analysed using unconditional and mixed-effects logistic regression, revealing a percentage of TiPV infection was 11.88%. Two significant risk factors associated with TiPV infection were identified: the source of the fish (*p*=0.020) and the initial fish weight at the stocking date (*p*=0.026). Conversely, the feeding method (*p*=0.039) was found to be a protective factor against TiPV infection. This study is the first to investigate the epidemiology of TiPV infection in farmed red hybrid tilapia. Our findings are important for improving farm management practices, mitigating the risk of TiPV infection and developing effective disease control strategies.

## 1. Introduction

Tilapia is the third most important farmed fish globally following grass carp (*Ctenopharyngodon idellus*) and silver carp (*Hypophthalmichthys molitrix*), respectively, with an annual production of 7.2 million tons and an estimated value of USD 14.1 billion per year. Tilapia farming plays an important role as an accessible, low-cost aquaculture option for small-scale farmers and an inexpensive source of protein for domestic consumption and export. Among the 45 countries that produce tilapia, China, Indonesia and Egypt are the major producers, with annual production figures of 1.78 million, 1.22 million and 0.88 million tons, respectively [1]. In Thailand, tilapia is the most important aquatic animal in terms of annual production at 269,394 tons and an estimated value of USD 14.1 billion per year [2]. The main species of tilapia cultured in Thailand are Nile tilapia (*Oreochromis niloticus*) and red hybrid tilapia (*Oreochromis* spp.).

Despite the adaptability of tilapia to a range of farming conditions, their rapid growth rate and ease of breeding, tilapia aquaculture has faced substantial challenges due to the emergence and re-emergence of infectious diseases in recent years, both in Thailand and globally [3–9]. Among the various bacterial and viral infections affecting tilapia, emerging viruses, such as tilapia lake virus (TiLV), tilapia parvovirus (TiPV) and infectious spleen and kidney necrosis virus (ISKNV), are causing high mortality and having a substantial economic impact on tilapia farming worldwide [4–11]. Notably, a recent study showed that TiPV in China has been associated with a high mortality rate of 60%–70% in cultured adult tilapia [11]. Additionally, coinfection with TiPV and TiLV has caused high mortality in juvenile red hybrid tilapia in river cages in Thailand [12].

Fish infected with TiPV often exhibit clinical signs such as lethargy, reduced feeding, discolouration, skin haemorrhages, exophthalmia, severe ocular lesions and unusual mortality. Infected fish may display abnormal swimming behaviours, such as staying near the water surface or exhibiting corkscrew movements [11]. Epidemiological data are important for understanding pathogen distribution in emerging diseases and are invaluable for planning disease control and prevention strategies [13–17]. However, epidemiological data on TiPV are limited worldwide. Notably, the prevalence of TiPV in the major tilapia-producing provinces in China has been reported to range from 22.60% to 64.60% [11]. Recently, a prevalence as high as 95% has been reported in farmed tilapia in the Uttar Pradesh region of India. Interestingly, coinfection with TiPV and TiLV has also been documented, with a prevalence of 59.60% and high mortality in affected farms [10]. Similarly, both TiPV and TiLV were detected in juvenile red hybrid tilapia cultured in river cages in Thailand, although the extent of their prevalence was not investigated [12]. Despite these studies, comprehensive knowledge regarding the epidemiology and risk factors associated with the spread of TiPV remains limited. This information is crucial for effectively controlling and preventing the spread of this novel virus. In this study, we aimed to investigate the distribution and risk factors associated with TiPV infection in red hybrid tilapia farms. By examining the percentage of TiPV infection, its geographic distribution and the characteristics and management practices of various farms, we sought to identify and address the knowledge gaps in TiPV epidemiology.

## 2. Materials and Methods

### 2.1. Study Design and Sample Collection

This cross-sectional study was conducted from September 2022 to March 2024 to cover a full cycle of fish farming activities over a single year. The number of samples required to assess the prevalence of TiPV in Thailand was based on an estimated prevalence of 20% and determined using Epitools software [11]. The sensitivity and specificity were set at 90% and 99%, respectively, with a precision of 0.1 and a 95% confidence interval (CI) level. The calculation resulted in an estimated sample size of 75. To ensure robust data collection, a total of 101 samples were collected. Each sample comprised five moribund red hybrid tilapia, obtained from 40 fish farms across various provinces in Thailand. Detailed information of the samples collected from each farm were presented in Supporting Information 1: Table S1.

The selected farms were grouped according to five different practices: floating cages in rivers, floating cages in earthen ponds, concrete ponds, polyethylene (PE)-lined ponds and earthen ponds. The sampling locations were focused on the central, northern, western and eastern regions of Thailand and specifically the provinces of Uttaradit, Nakhon Sawan, Uthai Thani, Chai Nat, Ang Thong, Phra Nakhon Si Ayutthaya, Nakhon Pathom, Kanchanaburi, Ratchaburi and Prachin Buri, where the majority of tilapia farms are located. We used a snowball sampling technique, in which we relied on information supplied by fish farmers, to select the farms for the sample collection. Farmers who observed sick or dead fish contacted the research team to arrange for sample collection. The criteria for sample selection were based on the assessment of fish populations with a mortality rate exceeding 20% within 1 week of the first signs of infection, which is considered an abnormal rate that requires investigation and treatment. The number of samples collected from each farm corresponded to the number of sets of sick fish, with one sample comprising a randomly selected five fish per set. Gill biopsies and skin scraping samples were examined for ectoparasites under light microscopy, bacterial isolation was performed using samples from the anterior kidney, and TiPV detection was conducted using TaqMan probe-based quantitative polymerase chain reaction (qPCR) in pooled spleen, kidney and liver samples. The presence of TiLV was evaluated using reverse transcription qPCR (RT-qPCR) performed on pooled liver samples. In addition, the water quality parameters were determined as follows: dissolved oxygen (DO) and temperature using a DO meter (Lutron, Taipei, Taiwan); ammonia and nitrite levels using a commercial water quality test kit (Greater Vet, Nonthaburi, Thailand); and pH using a pH meter (OHAUS, Shanghai, China). The water quality measurements were conducted once, on the date of sample collection. The animal use and handling protocol were approved by the Institutional Animal Care and Use Committee, Kasetsart University, under protocol number ACKU67-VET-046.

### 2.2. TiPV Detection Using TaqMan Probe-Based qPCR Assays

The total DNA was extracted from the pooled spleen, kidney and liver samples collected from five fish using a commercial DNA extraction kit (Invitrogen, Carlsbad, CA, USA) in line with the manufacturer's instructions. The quality and quantity of the DNA were evaluated using a NanoDrop 2000c UV-Vis Spectrophotometer (Thermo Fisher Scientific, Waltham, MA, USA). Briefly, the TaqMan probe-based quantitative PCR protocol included an initial denaturation step at 95°C for 2 min, followed by 40 cycles at 95°C for 5 s and 61.9°C for 30 s. The assay was performed using a CFX96 Touch Deep Well Real-Time PCR Detection System (BioRad, Hercules, CA, USA) with a TiPV-specific probe by applying a protocol described elsewhere [18].

### 2.3. Detection of TiLV Using RT-qPCR Assays

RNA was extracted from pooled liver samples collected from five fish using a commercial RNA extraction kit (GeneAll, Seoul, Korea) according to the manufacturer's instructions. The RNA quality and quantity were assessed using a NanoDrop 2000c UV-Vis Spectrophotometer (Thermo Fisher Scientific, Waltham, MA, USA), and the RNA concentration was adjusted to 200 ng/µL with molecular-grade water. First-strand cDNA synthesis was carried out using a RT kit (Vivantis, Selangor Darul Ehsan, Malaysia) under the following conditions, 65°C for 5 min, 42°C for 60 min and 85°C for 5 min, using a T100 thermal cycler (BioRad, Hercules, CA, USA). RT-qPCR was performed using a CFX96 Touch Deep Well Real-Time PCR Detection System (BioRad, Hercules, CA, USA) with iTaq Universal SYBR Green Supermix (BioRad, Hercules, CA, USA). The primers and protocol used were based on a previously described method [19]. The thermal cycling conditions consisted of an initial denaturation at 95°C for 3 min, followed by 40 cycles of 95°C for 10 s and 60°C for 30 s.

### 2.4. Farm Data Collection

Data relevant to the farm characteristics and management practices were collected through questionnaire surveys during face-to-face interviews with the farm owners, managers and veterinarians responsible for fish health and management and via direct observation by the research team (Supporting Information 2). Before the data collection, the research objectives were communicated to the participants, who provided signed consent forms to confirm their participation. All the data were kept confidential, including the farms' names and locations, the names of the involved participants and any identifying information about the farms with disease occurrences. The protocol for this study was approved by the Kasetsart University Research Ethics Committee (KUREC-KPS67/072) and was conducted in accordance with the approved protocol.

### 2.5. Epidemiological Investigation and Statistical Analysis

The data obtained from the tilapia farms were analysed to identify the factors associated with TiPV detection, including the farm characteristics, management practices and the presence of other pathogens, as detailed in Table 1. The data were compiled into an Excel spreadsheet (Microsoft Excel 2019 version, Redmond, WA, USA) and imported into the STATA program (version 14.2, Stata Corp, College Station, TX, USA) for statistical analysis. The descriptive statistics for the categorical variables were presented as frequencies and percentages, while the continuous variables were presented as means with 95% CI. TiPV infection status served as the dependent variable. The independent variables were grouped as follows:

1. Sources of water: Public (three major rivers) and private water sources (one farm used water from a private water conveyance canal)
2. Water systems: Open (no water treatment) and close containment (water treated through filtration and chemical processes before being recycled into culture ponds)
3. Hatcheries: Large-scale breeding hatcheries (producing over one million fish fry per day with robust genetic improvement programs) and small- to medium-scale breeding hatcheries (producing less than 500,000 fry per day and relying on domestic parent brood stock)
4. Weather conditions: Divided into quarters—Q1 (January to March, cool weather and no rainfall), Q2 (April to June, hot weather and occasional rainfall), Q3 (July to September, rainy season) and Q4 (October to December, decreased rainfall and declining temperatures)
5. Fish carcass management: Efficient management (proper burial, incineration and composting) and poor management practices (discarding into public water or feeding to other fish/animals)
6. Initial weight of fish at stocking date: Analysed using locally weighted regression (lowess smoothing) with a discontinuous relationship found at 23 g. Data divided into two categories: initial weights ≤ 23 and >23 g

**Figure 1:**
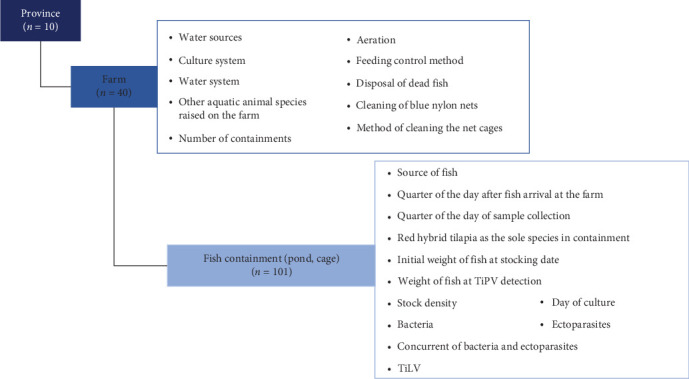
Cluster of the data used in the analysis. The data were hierarchically clustered at two levels, with each level showing the number of data records.

For the statistical analysis and risk factor identification, the data were organised into a two-level hierarchical structure to reflect the farm and province levels, as shown in Figure 1. The association between TiPV infection and each predictor variable was assessed using mixed-effects logistic regression. In this study, the provinces were not randomly selected, and there were no predictors at province level; thus, the province was included as a fixed effect. In contrast, the predictor variables at farm level varied based on the different farm characteristics and management practices, so the farm was classified as a random effect. Before conducting the mixed-effects logistic regression to analyse the risk factors, we applied unconditional logistic regression to identify the variables with *p*-values < 0.1 as potential predictors. Mixed-effects logistic regression analysis was subsequently performed using these potential predictors. Moreover, the culture system was identified as a confounding variable due to its influence on many of the analysed factors. It was therefore included in the analysis to account for its association with TiPV infection and the potential predictors. The percentage change in the odds ratios (ORs) before and after accounting for these confounders was evaluated. The results were presented as OR, standard error (SE), standard normal deviate (*Z*), 95% CI and *p*-value. A factor with an OR > 1 and a *p*-value < 0.05 was considered a risk factor, while a factor with an OR < 1 and a *p*-value < 0.05 was deemed a protective factor.

## 3. Results

### 3.1. General Information

**Figure 2:**
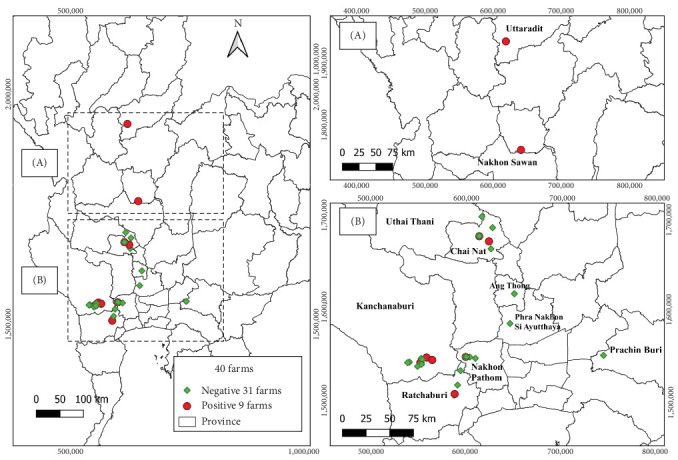
Geographic distribution of tilapia parvovirus (TiPV) infection in the red hybrid tilapia-positive farms: positive farms are indicated by red circles and negative farms by green diamond-shaped quadrangles. (A) Distribution in the upper central and northern regions. (B) Distribution in the western, central and eastern regions. The map was generated using QGIS version 3.32.

Between September 2022 and March 2024, a total of 101 samples, each comprising five fish, were processed for TiPV detection. These samples were collected from 40 distinct farms located in the central, eastern, western and northern regions of Thailand (Figure 2).

**Figure 3:**
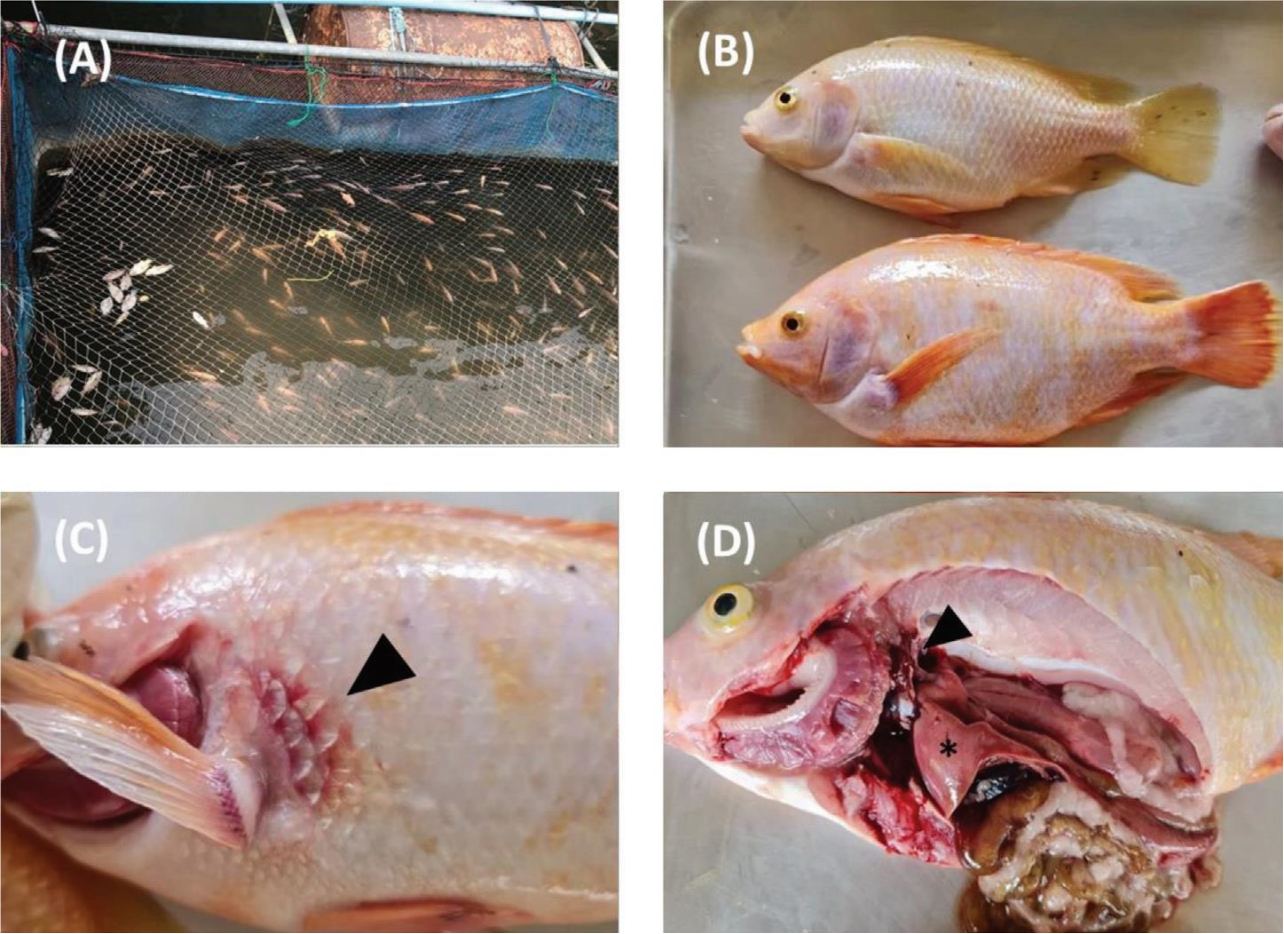
(A) Dead fish were observed in a floating cage in a river. (B) External appearance of diseased fish showing the pale colour of the bodies. (C) Haemorrhage or congestion at the base of the pectoral fin (black arrow). (D) Necropsy findings revealed discolouration and paleness in the liver (asterisk) and an enlarged kidney (black arrow).

The inclusion criteria of the farms were those with a history of abnormal mortality characterised by a mortality rate exceeding 20% within one week of the first signs of infection. The weight of the sampled fish ranged from 0.5 to 1000 g. TiPV was detected in the fish samples from 9 of the 40 farms. Among these, eight farms had fish in cages in rivers, while one farm had fish in both river cages and concrete ponds. The diseased fish showed cumulative mortality rates ranging from 30% to 80%. The clinical signs observed in the diseased fish included anorexia, lethargy, swimming near the water surface, pale skin and haemorrhages at the base of the fins and tail and on the body surface. Further necropsy revealed that the fish had pale livers; enlarged kidneys, spleens and gall bladders; and empty intestines (Figure 3).

During the ectoparasite examination of the diseased fish, various types of parasites were identified: *Trichodina* spp. in 57 out of 101 samples (56.44%), *Dactylogyrus* spp. in 47 samples (46.53%), *Gyrodactylus* spp. in 25 samples (24.75%), *Ichthyobodo* spp. and *Scyphidia* spp. in five samples (4.95%), *Chilodonella* spp. in four samples (3.96%), *Myxobolus* spp. in one sample (0.99%) and no ectoparasite detected in nine samples (8.91%) as detailed in Table 2 and Supporting Information 1: Table S1. Moreover, common pathogenic bacteria were detected, namely, *Aeromonas* spp. in 90 samples (89.10%), *Streptococcus* spp. in 52 samples (51.49%), *Flavobacterium* spp. in 14 samples (13.86%) and *Francisella* spp. in two samples (1.98%). No bacterial growth was found in three samples (2.97%), as detailed in Table 2 and Supporting Information 1: Table S1.

The water quality parameters were recorded as temperatures that ranged from 26.5 to 28°C, pH from 7.6 to 8, DO levels from 2.7 to 9 ppm, total ammonia from 0 to 1 ppm and nitrite levels from 0.1 to 0.3 ppm.

### 3.2. Percentages of TiPV Infection and Geographic Distribution

The percentage of TiPV infection was 11.88%, with 12 of 101 sampled fish testing positive. Among the positive fish farms, three were located in Kanchanaburi province (western region), two in Chai Nat province, one in Nakhon Pathom, one in Ratchaburi (central region), one in Nakhon Sawan and one in Uttaradit province (northern region) (Figure 2). Notably, these nine farms were located along the two of the main rivers for fish farming in Thailand, which are also primarily public water sources: a tributary of the Mae Klong (Chai Nat, Kanchanaburi and Nakhon Pathom) and a tributary of the Chao Phraya River (Chai Nat, Nakhon Sawan and Uttaradit).

### 3.3. Detection of TiLV in Fish Samples

Due to the similarity in external clinical signs and lesion between TiLV and TiPV infections, we tested for the presence of TiLV on 84 of the 101 samples. The remaining samples collected during the early phase of the study were not tested for TiLV. Notably, none of these 17 samples tested positive for TiPV. Among the 84 samples analysed, 40 were found to be infected with TiLV. Of these, 31 showed single TiLV infections, three showed single TiPV infections, and nine were identified as TiPV–TiLV coinfections (Supporting Information 1: Table S1). Although the clinical signs of single TiPV infections and TiPV–TiLV coinfections were indistinguishable, differences in cumulative mortality rates were observed. Specifically, single TiPV infections were associated with cumulative mortality rates of 30%–50%, while the coinfections with TiLV resulted in significantly higher rates, ranging from 30% to 80%.

### 3.4. Descriptive Statistics and Unconditional Logistic Regression Analysis of the Farm Characteristics and Management Practices

The descriptive statistics showing comparisons between the TiPV infection and noninfection groups as well as the results of the unconditional logistic regression analysis are presented in Table 3. Six factors were associated with TiPV infection (*p* < 0.1):

1. Water sources: The farms along the Mae Klong had eight positive samples out of 83 (9.64%), while those along the Chao Phraya River had four positive samples out of 13 (30.77%). No TiPV-positive samples were found for the farms that used water from the Bang Pakong River or water conveyance canals.
2. Fish source: TiPV detection was significantly linked to the large-scale breeding hatcheries. Specifically, hatchery source no. 1 had three positive samples out of 11 (27.27%), and hatchery no. 3 had three positive samples out of seven (42.86%).
3. Feeding control method: The farms that used the ad libitum feeding method had 10 positive samples out of 52 (19.23%) compared to only two out of 49 (4.08%) for the farms that used feed intake relative to fish body weight.
4. Initial weight of the fish at the stocking date: The fish weighing more than 23 g had a significantly higher rate of TiPV infection (10 out of 40 samples, 25%) compared to those that weighed 23 g or less (two out of 61 samples, 3.28%).
5. The weight of the fish at TiPV detection: The infected fish had a significantly higher average weight of 336.95 g (range, 95.60–578.31 g).
6. TiLV detection: Of the 12 samples that tested positive for TiPV, nine (75%) were identified as coinfected with TiLV.

All six factors identified as potential risk factors for TiPV infection were further analysed using mixed-effects logistic regression analysis. Some factors, including the water system and coculture with other species, could not be analysed to determine the *p*-values due to the absence of infected fish under these conditions. When these factors were adjusted to include positive samples across all conditions, no statistically significant differences were observed (*p* > 0.1). We analysed the association between TiPV infection and other pathogens, including ectoparasites, bacteria and concurrent infections involving multiple pathogens. However, this analysis could not be performed because all TiPV-positive samples had coinfections with bacteria and ectoparasites, preventing the calculation of a *p*-value. To address this limitation, an additional analysis was conducted by simulating a scenario in which one TiPV-positive sample lacked coinfections. Despite this adjustment, the statistical analysis continued to yield a *p*-value that were not significantly different.

### 3.5. Mixed-Effects Logistic Regression Analysis

The mixed-effects logistic regression analysis identified three statistically significant factors (*p* < 0.05) in the final model: two risk factors and one protective factor (Table 4). The first risk factor was the initial weight of the fish at stocking: the fish weighing more than 23 g were 18 times more likely to be detected with TiPV infection (OR = 18.00). The second risk factor was the fish source. We found that the fish from the large-scale breeding hatcheries had a higher risk of infection compared to those from small- to medium-scale breeding hatcheries. For example, the infection risk for fry from hatchery source no. 1 was up to 67 times higher (OR = 67.36). Conversely, the feeding control method was identified as a protective factor, as applying the feed intake relative to fish body weight significantly reduced the likelihood of TiPV infection (OR = 0.08). Furthermore, the culture system, which was initially expected to be a confounding factor, was included in the analysis. However, no significant changes were observed in the OR or *p*-values, which indicated that there were no confounding factors affecting the results.

## 4. Discussion

The emergence of TiPV poses a serious threat to tilapia aquaculture and has led to substantial economic losses for farmers. Understanding the epidemiology and clinical manifestations of TiPV is important for the development of effective management and control strategies. In this study, we investigated the abnormal mortality of red hybrid tilapia in various regions of Thailand, with a specific focus on the percentage and distribution of TiPV and farm-related factors associated with TiPV infection. Notably, the infected fish showed cumulative mortality rates ranging from 30% to 80%, with nonspecific clinical signs, such as anorexia, lethargy, swimming near the water surface, pale skin and haemorrhages at the base of the fins and tail and on the body surface. The necropsy findings revealed pale livers; enlarged kidneys, spleens and gall bladders; and empty intestines, which is consistent with previous reports of TiPV in China [11]. Contrary to the findings of the study by Liu et al. [11] in which TiPV infection in China only affected adult tilapia, we found that TiPV affected all age groups of red hybrid tilapia, from fry to adults. Specifically, the smallest infected fish weighed 3.33 g, while the largest weighed 1027.33 g. Interestingly, coinfections with parasites and bacteria, which can contribute to higher mortality rates, were detected in all the TiPV-infected fish. The farming practices in Thailand, where fish of different ages are reared together and open water systems are commonly used, likely facilitated these coinfections [12]. We further found that 11.88% (12 out of 101) of the samples tested positive for TiPV, which represented a lower detection rate than those in reports from China (22.60%–64.60%) [11] and India (59.60%–95.00%) [10]. Among the 12 TiPV-positive samples, nine were coinfected with both TiPV and TiLV. While the clinical signs of single TiPV infections and TiPV–TiLV coinfections were indistinguishable, notable differences in cumulative mortality rates were observed. Single TiPV infections had cumulative mortality rates of 30%–50%, consistent with a previous TiPV study in China [11]. In contrast, coinfections with TiLV resulted in higher cumulative mortality rates, ranging from 30% to 80%, which aligns with a previous report on TiPV and TiLV coinfections in river cage systems [12]. Several factors could have contributed to these differences, including variations in the severity of the outbreaks, differences in farming practices, the sources of the fry and fingerlings and environmental conditions in the culture areas. The higher prevalence of TiPVin China and India may account for the more severe outbreaks or higher transmission rates of TiPV in those regions. Factors such as the stock density of fish farms, the greater movement of fish between farms and the intensity of aquaculture practices could also influence the spread and impact of this virus. Nonetheless, the percentage of TiPV infection in this study was not derived from a random sampling process, so direct comparisons of prevalence between related studies was not possible. To obtain a more accurate estimate of TiPV prevalence in Thailand, future studies should include random sampling across a broader geographic area; our samples were mainly acquired from the central and western parts of Thailand due to transportation limitations and a lack of mortality reports from farmers in other regions.

In our analysis of the risk factors associated with TiPV infection, a significant risk factor was identified as the source of the fish, notably those sourced from large-scale breeding hatcheries. Screening is likely not yet routinely conducted for TiPV before introducing new fish or moving fish within hatchery farms, as it is an emerging pathogen. Consequently, the possibility exists that the virus could spread within some hatcheries and subsequently be transmitted to other aquaculture areas and facilities [20]. This hypothesis is supported by the fact that small-scale farmers in Thailand who raise tilapia in open water systems often acquire fingerlings from different hatcheries. Additionally, the lack of disease screening for emerging pathogens, like TiPV, combined with the practice of rearing fish at high stock densities, which promotes the spread of pathogens and stresses fish, could increase the contact rates between fish and accelerate the transmission of the virus [20]. To address these challenges, future efforts should focus on implementing routine screening for TiPV in hatcheries, improving genetic resistance traits in breeding programs and adopting farming practices that minimise disease transmission. Such key strategies include maintaining optimal stocking densities to reduce fish stress and regularly disinfecting equipment and cages, both of which can remarkably decrease susceptibility to TiPV and other infections.

The second risk factor for TiPV infection is the use of fish with an initial weight greater than 23 g. Typically, small-scale farmers transport fingerlings weighing more than 20 g from hatcheries to grow-out cages in rivers. These fingerlings are initially sourced from hatcheries as fry that weigh 0.5–2 g and reared in ponds or cages in ponds until they reach ~20–30 g. The practice of using fingerlings from multiple hatcheries, combined with the presence of TiPV in some grow-out areas, can lead to contamination and the transmission of the virus. When these fish are raised together in the same environment without quarantine measures, TiPV can spread. Additionally, the fish tanks and vehicles used to transport fingerlings, which are often not properly cleaned and disinfected, can contribute to the risk of transmission. These same fish tanks are also commonly used to transport larger live fish to market, which further increases the potential for virus transmission. Previous studies undertaken in China and India support this supposition, as TiPV has frequently been detected in larger tilapia [10, 11].

Given that the TiPV infection was higher in the farms that used water from the tributary of the Chao Phraya River (3.96%) and the tributary of the Mae Klong (7.92%), it is important to highlight that all the positive samples came from farms that utilised open water systems. Although this finding was not statistically significant, it suggests that open water systems facilitate waterborne TiPV transmission or infection through wild fish carriers [21]. In contrast, no infections were detected in the samples from the farms that used closed water systems. In the latter type of system, the water is filtered and treated with disinfectants, such as chlorine, before use. The effectiveness of common disinfectants against TiPV remains to be determined. However, previous studies have demonstrated that most common disinfectants in tilapia farms can inhibit emerging viruses, such as TiLV [22]. Despite the small number of farms in our study that used closed water systems, which was insufficient to demonstrate the effectiveness of the system as a protective factor, the observed trend suggests the potential role of farming practices in the spread of TiPV. This warrants further investigation with larger sample sizes to draw more definitive conclusions. Moreover, water quality parameters in open water systems are highly dynamic and subject to continuous fluctuation. Identifying these parameters as potential risk factors requires longitudinal studies conducted across diverse locations, incorporating continuous monitoring through real-time measurement devices. Such approaches would facilitate tracking water quality changes over time and enable retrospective analyses during outbreaks to identify abnormal parameters associated with disease emergence.

In addition to the previously mentioned risk factors, other variables that may be associated with TiPV detection include the presence of other species within the same environments as tilapia. These species include climbing perch (*Anabas testudineus*), walking catfish (*Clarias* spp.), Siamese pangasius (*Pangasius macronema*), carp (*Cyprinus carpio*) and red-tailed tinfoil (*Barbonymus altus*). Tilapia may acquire the virus from wild fish that act as carriers, such as red-tailed tinfoil, which can enter cages because they are smaller than the net mesh size. Although the ability of these other fish species to act as carriers of TiPV has not been confirmed, studies on other emerging tilapia viruses, such as TiLV, have shown that the virus can infect other freshwater fish species [23]. TiPV has also been detected in the faeces of crocodiles that consumed tilapia [24]. While it has not been confirmed whether the virus remains viable and infectious in this context, this evidence suggests that other animals could potentially play a role in the spread of TiPV. Interestingly, a study demonstrated that Indian carp (*Catlacatla*) and rohu (*Labeorohita*) that cohabited with TiPV-infected tilapia did not show clinical signs of infection, and the virus could not be detected by molecular assay [25]. To verify whether other fish species are susceptible to infection, future research should involve testing for TiPV in fish that live in the same environment as red hybrid tilapia. Another finding from our study is that the month and season that the fish were stocked and the samples collected had no impact on the detection of TiPV, as the virus was found throughout the year, which is similar to TiLV detection [26]. Nevertheless, this is in contrast to other important bacterial infections, such as francisellosis, which typically occurs during cooler months [27], and streptococcosis, which is more prevalent during hotter months [28, 29]. Regarding the ectoparasites, bacteria and their concurrent presence identified in the samples analysed in this study, no significant association was found between these pathogens and TiPV infection. Still, the presence of ectoparasites and bacteria or their concurrence with TiPV infection could contribute to heightened mortality rates and accelerated disease progression, depending on predisposing factors or pathogen load under specific rearing conditions [30]. Additionally, we found frequent coinfections between TiPV and TiLV, which may contribute as a potential risk factor exacerbating the severity of TiPV infection outcomes. However, the final model including TiLV was compared to the model without TiLV using log-likelihood analysis, which indicated that the model excluding TiLV was more appropriate for our data.

Based on the analysis of the various factors, our results indicated that the percentage of feed intake relative to fish body weight is an important protective factor. This method effectively reduces the risk of disease in farms by ensuring that the fish receive a consistent and appropriate amount of feed daily. In contrast, ad libitum feeding results in large fluctuations in daily feed intake, with fish potentially receiving either excessive or insufficient amounts of feed. Overfeeding can lead to bloating and consequent stress [31], while underfeeding can weaken fish, which compromises their immune systems and increases their susceptibility to infection [32]. Additionally, leftover feed may promote the accumulation of biofouling, such as plants, algae or small animals, which may cause net clogging, reduce water flow and lead to the accumulation of waste inside cages [33]. Providing an appropriate amount of feed not only helps reduce waste accumulation in the farm environment but also lowers production costs, as feed costs account for more than 70% of total costs [34].

Given that the key risk factors associated TiPV infection identified in this study were the source of fingerlings and their weight at the time of stocking, farmers should ensure they use fingerlings from reputable sources that have effective disease management protocols in place. It is also important to screen all batches of fingerlings for specific pathogens before stocking. Farmers should consider raising their own fingerlings to the desired size to avoid sourcing fish from multiple locations. Importantly, implementing a robust biosecurity system is essential to reduce the risk of pathogen entry into farms. In addition to the three previously mentioned factors, another recommended practice is to clean the blue nylon nets during the production cycle to improve overall farm hygiene and water flow and thereby reduce pathogen accumulation. Rearing fish in a closed water system is also a promising practice for preventing TiPV introduction and transmission. However, we found that the number of farms that cleaned the blue nylon nets during the production cycle and used closed water systems was low, which resulted in no statistically significant association with TiPV infection. In future research, a case–control study should be conducted to verify this association.

## 5. Conclusion

Epidemiological studies and the identification of risk factors associated with the spread of viruses are crucial for the development of effective strategies to mitigate and prevent TiPV infection among tilapia populations. In this study, we found an 11.88% TiPV infection rate among farmed red hybrid tilapia in Thailand. We further identified two significant risk factors associated with TiPV infection: the source of the fish and the initial weight of the fish at the time of stocking. In contrast, the feeding method based on a percentage of fish body weight was identified as a protective factor. These findings are valuable for guiding disease management efforts aimed at mitigating the proliferation of this emerging virus within tilapia farming systems.

## Figures and Tables

**Table 1:** Explanatory variables.

Variables	Description
Dependent variable	
TiPV infection status	A mortality rate exceeding 20% within 1 week of the first signs of infection and confirmation of virus presence by TaqMan qPCR
Independent variables	
Farm characteristics	
Farm location	The province where the farm was located
Water sources	Water source used on the farm, including public (e.g. a tributary of the Chao Phraya River, a tributary of the Mae Klong and the Bang Pakong River) and private water sources (water conveyance canals)
Culture system	Type of red hybrid tilapia culture
Water system	Whether water treatments or reservoir water was applied before adding to the fish containments
Other aquatic animal species	Other aquatic species raised on the farm
Number of containments	Number of net cages or ponds on the farm
Farm management practices	
Source of fish	Hatcheries that provided fish seeds
Quarter of the day after the fish arrival	The time of fish stocking into the containments
Quarter of the day of sample collection	The time of sample collection
Aeration	Whether the farm operated with or without aerators
Feeding control method	Methods for controlling the feeding rate, namely, the percentage of feed intake relative to fish body weight and ad libitum feeding, where fish consume food in line with their appetite. This results in high daily fluctuations in feed intake rates, with no upper limit set as a percentage of body weight
Disposal of dead fish	Methods used in farm practices for the disposal of dead fish
Cleaning of blue nylon nets	Whether the blue nylon nets (inner mesh to prevent feed pellets from escaping) were cleaned during the production cycle
Method of cleaning the net cages	Methods used for cleaning the net cages, that is, washing with high-pressure water, sun drying, combining both methods or applying disinfectant after washing with high-pressure water and sun drying
Red hybrid tilapia as the sole species in containment	Whether red hybrid tilapia was the only species in the containment or there was coculture with other species
Day of culture	Duration of stocking until date of sample collection (days)
Initial weight of fish at stocking date	Body weight of fish at stocking date (g)
Weight of fish at TiPV detection	Body weight of fish at the sampling date (g)
Stock density	Number of fish stocked into the fish containments (fish/m^3^)
The presence of pathogens	
Ectoparasites	The presence or absence of ectoparasites under light microscopy
Bacteria	The presence or absence of bacteria through bacterial isolation from anterior kidney
Concurrent infections by multiple bacteria and ectoparasites	The presence of multiple infections involving more than one species of bacteria and ectoparasites within a single sample
TiLV	The presence or absence of TiLV was confirmed by RT-qPCR

Abbreviations: RT-qPCR, reverse transcription quantitative polymerase chain reaction; TiLV, tilapia lake virus; TiPV, tilapia parvovirus.

**Table 2:** Summary of ectoparasites and bacteria detected in the samples.

Ectoparasites*⁣*^*∗*^	%	Bacteria*⁣*^*∗*^	%
*Trichodina* spp.	56.44	*Aeromonas* spp.	89.10
*Dactylogyrus* spp.	46.53	*Streptococcus* spp.	51.49
*Gyrodactylus* spp.	24.75	*Flavobacterium* spp.	13.86
*Ichthyobodo* spp.	4.95	*Francisella* spp.	1.98
*Scyphidia* spp.	4.95	No bacterial growth	2.97
*Chilodonella* spp.	3.96	—	—
*Myxobolus* spp.	0.99	—	—
No parasite detected	8.91	—	—

*⁣* ^*∗*^Some samples were found to contain multiple bacteria or ectoparasites.

**Table 3:** Descriptive statistics and unconditional logistic regression analysis of the farm characteristics, management practices and the presence of other pathogens.

Factors	TiPV negative			TiPV positive			
	*N*	%	Mean (95% CI)	*N*	%	Mean (95% CI)	*p*-Value
Farm characteristics							
Farm location							
Chai Nat	15	16.85	—	2	16.67	—	1
Uttaradit	3	3.37	—	1	8.33	—	0.506
Nakhon Sawan	0	0	—	2	16.67	—	—
Uthai Thani	2	2.25	—	0	0	—	—
Ang Thong	1	1.12	—	0	0	—	—
Phra Nakhon Si Ayutthaya	1	1.12	—	0	0	—	—
Nakhon Pathom	13	14.61	—	1	8.33	—	0.688
Kanchanaburi	40	44.94	—	5	41.67	—	0.942
Ratchaburi	13	14.61	—	1	8.33	—	0.688
Prachin Buri	1	1.12	—	0	0	—	—
Water source							
A tributary of the Chao Phraya River	9	10.11	—	4	33.33	—	1
A tributary of the Mae Klong	75	84.27	—	8	66.67	—	0.043
Bang Pakong River	1	1.12	—	0	0	—	—
Water conveyance canal	4	4.49	—	0	0	—	—
Culture system							
Floating cages in the river	77	86.52	—	11	91.67	—	1
Concrete ponds	4	4.49	—	1	8.33	—	0.631
Floating cages in earthen pond	1	1.12	—	0	0	—	—
Earthen ponds	6	6.74	—	0	0	—	—
Polyethylene-lined pond	1	1.12	—	0	0	—	—
Water system*⁣*^*∗*^	—	—	—	—	—	—	–
Open containment	81	91.01	—	12	100.00	—	—
Close containment	8	8.99	—	0	0	—	—
Other aquatic animal species raised on a farm	—	—	—	—	—	—	0.507
Yes	14	15.73	—	1	8.33	—	—
No	75	84.27	—	11	91.67	—	—
Number of containments	—	—	105.26 (83.86–126.66)	—	—	114.25 (56.37–172.13)	0.769
Farm management practices							
Source of fish							
Small- to medium-scale breeding hatcheries	28	31.46	—	2	16.67	—	1
Large-scale breeding hatcheries no. 1	8	8.99	—	3	25.00	—	0.096
Large-scale breeding hatcheries no. 2	26	29.21	—	2	16.67	—	0.943
Large-scale breeding hatcheries no. 3	4	4.49	—	3	25.00	—	0.026
Large-scale breeding hatcheries no. 4	8	8.99	—	1	8.33	—	0.664
Large-scale breeding hatcheries no. 5	15	16.85	—	1	8.33	—	0.957
Quarter of the day after the fish arrival							
Q1 (January to March)	19	21.35	—	2	16.67	—	1
Q2 (April to June)	20	22.47	—	3	25.00	—	0.714
Q3 (July to September)	23	25.84	—	1	8.33	—	0.484
Q4 (October to December)	27	30.34	—	6	50.00	—	0.390
Quarter of the day of sample collection							
Q1 (January to –March)	30	33.71	—	6	50.00	—	1
Q2 (April to June)	16	17.98	—	2	16.67	—	0.590
Q3 (July to September)	16	17.98	—	2	16.67	—	0.590
Q4 (October to December)	27	30.34	—	2	16.67	—	0.247
Aeration	—	—	—	—	—	—	0.241
Aerator used	43	48.31	—	8	66.67	—	—
No aerator used	46	51.69	—	4	33.33	—	—
Feeding control method	—	—	—	—	—	—	0.032
Ad libitum	42	47.19	—	10	83.33	—	—
% feed intake relative to fish body weight	47	52.81	—	2	16.67	—	—
Disposal of dead fish	—	—	—	—	—	—	0.371
Efficiency	63	70.79	—	10	83.33	—	—
Inefficiency	26	29.21	—	2	16.67	—	—
Cleaning of blue nylon nets (89 samples)	—	—	—	—	—	—	0.132
Cleaning during the production cycle	13	16.67	—	4	36.36	—	—
No cleaning during the production cycle	65	83.33	—	7	63.64	—	—
Method of cleaning the net cage (89 samples)							
Wash with high-pressure water	27	34.62	—	5	45.45	—	1
Sun dry	4	5.13	—	1	9.09	—	0.806
Wash with high-pressure water and sun dry	38	48.72	—	3	27.27	—	0.270
Washing with high-pressure water, disinfectant and sun dry	9	11.54	—	2	18.18	—	0.843
Red hybrid tilapia the sole species in the containment*⁣*^*∗*^	—	—	—	—	—	—	–
Yes	84	94.38	—	12	100.00	—	—
No	5	5.62	—	0	0	—	—
Day of culture	—	—	77.28 (64.64–89.92)	—	—	80.75 (49.82–111.68)	0.847
Initial weight of fish at stocking date	—	—	—	—	—	—	0.005
≤23 g	59	66.29	—	2	16.67	—	—
>23 g	30	33.71	—	10	83.33	—	—
Weight of fish at TiPV detection (g)	—	—	192.48 (142.78–242.17)	—	—	336.95 (95.60–578.31)	0.072
Stock density (fish/m^3^)	—	—	198.56 (101.41–295.70)	—	—	247.65 (−203.05–698.340)	0.746
The presence of other pathogens*⁣*^*∗∗*^							
Ectoparasites (95 samples)	—	—	—	—	—	—	–
Negative	9	10.84	—	0	0	—	—
Positive	74	89.16	—	12	100.00	—	—
Bacteria	—	—	—	—	—	—	–
Negative	3	3.37	—	0	0	—	—
Positive	86	96.63	—	12	100.00	—	—
Bacterial and ectoparasitic concurrence (95 samples)	—	—	—	—	—	—	–
Negative	11	13.25	—	0	0	—	—
Positive	72	86.75	—	12	100.00	—	—
TiLV (84 samples)	—	—	—	—	—	—	0.052
Negative	41	56.94	—	3	25.00	—	—
Positive	31	43.06	—	9	75.00	—	—

Abbreviations: CI, confidence interval; TiLV, tilapia lake virus; TiPV, tilapia parvovirus.

*⁣* ^*∗*^A *p*-value could not be analysed due to the absence of TiPV-positive samples under certain conditions.

*⁣* ^*∗∗*^A *p*-value could not be analysed because all TiPV-positive samples were coinfected with bacteria and ectoparasites.

**Table 4:** Analysis of the mixed-effects logistic regression of the risk factors associated with TiPV infection and the source of fish.

Risk factors	OR	SE	*Z*	*p*-Value	95% CI
Initial weight of fish at stocking date	18.00	23.31	2.23	0.026	1.42–227.70
Source of fish					
Small- to medium-scale breeding hatcheries	—	—	—	1	—
Large-scale breeding hatchery no.1	67.36	121.41	2.34	0.020	1.97–2304.79
Large-scale breeding hatchery no. 2	1.03	1.14	0.03	0.979	0.12–9.07
Large-scale breeding hatchery no. 3	12.33	16	1.94	0.053	0.97–156.78
Large-scale breeding hatchery no. 4	20.44	37.31	1.65	0.098	0.57–731.30
Large-scale breeding hatchery no. 5	1.05	1.43	0.04	0.969	0.07–15.05
Feeding control method	0.08	0.10	−2.06	0.039	0.01–0.90

Abbreviations: CI, confidence interval; OR, odds ratio; SE, standard error; TiPV, tilapia parvovirus; *Z*, standard normal deviate.

## Data Availability

The data that support the findings of this study are available from the corresponding authors upon reasonable request.

## Supporting Information

Supporting Information 1 Table S1: Details of samples from tilapia farms and results of pathogen detection

Supporting Information 2 Questionnaire in English and Thai.
