# Supplementary material for: Distribution and Risk Factors Associated With Tilapia Parvovirus (TiPV) Presence in Red Hybrid Tilapia (Oreochromis spp.) Farms in Thailand
Source: Transbound Emerg Dis. 2025 Feb 10;2025:6618755. doi: 10.1155/tbed/6618755 (PMC12016865; doi:10.1155/tbed/6618755)
Supplement: Supporting Information 1 — Table S1: Details of samples from tilapia farms and results of pathogen detection [file 6618755.f1.docx]

**Supplementary Table S1:** Details of samples from tilapia farms and results of pathogen detection

| **Farm ID** | **No. of samples** | **Sample**  **ID** | **Ectoparasites** | **Bacteria** | **TiLV** | **TiPV** |
| --- | --- | --- | --- | --- | --- | --- |
| 1 | 1 | 1 | G | A, S, Fl | − | − |
| 2 | 2 | 2 | G | A | + | + |
|  |  | 3 | G | A, S, Fl | ND | − |
| 3 | 1 | 4 | D | A | − | − |
| 4 | 3 | 5 | D | A, S, Fl | + | + |
|  |  | 6 | − | A, S | ND | − |
|  |  | 7 | Ich | A, S | − | − |
| 5 | 1 | 8 | − | A, S | ND | − |
| 6 | 1 | 9 | G, T | A | + | − |
| 7 | 2 | 10 | T | A | − | − |
|  |  | 11 | ND | A, S | − | − |
| 8 | 1 | 12 | − | No growth | + | − |
| 9 | 4 | 13 | − | A | − | − |
|  |  | 14 | D | A | − | − |
|  |  | 15 | D, G | A | − | − |
|  |  | 16 | D | A | − | − |
| 10 | 1 | 17 | − | A, S | + | − |
| 11 | 5 | 18 | D | A | ND | − |
|  |  | 19 | G | A, S | ND | − |
|  |  | 20 | D, T | A, S | ND | − |
|  |  | 21 | Ich | A, S | ND | − |
|  |  | 22 | G | A, S | ND | − |
| 12 | 10 | 23 | D, T | A | − | − |
|  |  | 24 | D, T | A | ND | − |
|  |  | 25 | G, T | A | ND | − |
|  |  | 26 | D | A | ND | − |
|  |  | 27 | T, Ich | A, S | ND | − |
|  |  | 28 | T | A, S | ND | − |
|  |  | 29 | G, T | A, S | − | − |
|  |  | 30 | T | A, S | − | − |
|  |  | 31 | T | A, S | + | − |
|  |  | 32 | G | A, Fr | + | − |
| 13 | 1 | 33 | − | A | + | − |
| 14 | 2 | 34 | − | S | − | − |
|  |  | 35 | D | A, Fr | − | − |
| 15 | 1 | 36 | T | S | ND | − |
| 16 | 1 | 37 | ND | A, S | + | − |
| 17 | 3 | 38 | G | S | + | + |
|  |  | 39 | D, T | S | + | + |
|  |  | 40 | D, T | A, S | − | − |
| 18 | 3 | 41 | ND | A, S | − | − |
|  |  | 42 | G, T | A, Fr | + | − |
|  |  | 43 | G, T | A, S | − | − |
| 19 | 1 | 44 | ND | A | + | − |
| 20 | 13 | 45 | T | A | + | + |
|  |  | 46 | G, T | A | + | − |
|  |  | 47 | G, T | A | + | − |
|  |  | 48 | D, T | A | − | − |
|  |  | 49 | D, T | A | + | − |
|  |  | 50 | D, T | A | + | − |
|  |  | 51 | − | A | − | − |
|  |  | 52 | T | A | − | − |
|  |  | 53 | T | A, S | + | − |
|  |  | 54 | D, T | A | ND | − |
|  |  | 55 | D, G, T | A, S | ND | − |
|  |  | 56 | D, T | A | + | − |
|  |  | 57 | D, T | A | + | + |
| 21 | 3 | 58 | D, T | A, S | ND | − |
|  |  | 59 | D, G, M | A | + | + |
|  |  | 60 | G, T | No growth | − | − |
| 22 | 2 | 61 | D | A, S, Fl | − | − |
|  |  | 62 | D | A, S, Fl | − | − |
| 23 | 2 | 63 | D, T | A | − | − |
|  |  | 64 | G, T | A, S | − | − |
| 24 | 3 | 65 | D, T | A, S, Fl | + | − |
|  |  | 66 | D | A, Fl | + | − |
|  |  | 67 | D, T | A, S | − | − |
| 25 | 2 | 68 | G | A, S | − | − |
|  |  | 69 | D, T | A, S | − | − |
| 26 | 2 | 70 | T, Scy | A, S | − | − |
|  |  | 71 | T | A, Fl | + | + |
| 27 | 1 | 72 | G, T, Ich | A, S | − | − |
| 28 | 1 | 73 | D, G, T, Scy | No growth | − | − |
| 29 | 2 | 74 | D, T | A | + | − |
|  |  | 75 | T | A | + | − |
| 30 | 1 | 76 | G, T | A, S | + | − |
| 31 | 1 | 77 | ND | A, S | − | − |
| 32 | 4 | 78 | ND | A | + | − |
|  |  | 79 | T | A, S | + | + |
|  |  | 80 | T | A, S | + | − |
|  |  | 81 | ND | A, S | + | − |
| 33 | 2 | 82 | − | A, S | − | − |
|  |  | 83 | Ich | A, Fl | + | − |
| 34 | 1 | 84 | D, G | A, S, Fl | + | − |
| 35 | 1 | 85 | T | Fl | + | − |
| 36 | 5 | 86 | D, G, Scy | A | + | − |
|  |  | 87 | D, T, Scy | A | + | − |
|  |  | 88 | D, T, Scy, Ch | A | + | − |
|  |  | 89 | D, T, Ch | A, Fl | + | − |
|  |  | 90 | D, T, Ch | A, Fl | + | − |
| 37 | 2 | 91 | D, T, Ch | A, S, Fl | − | − |
|  |  | 92 | D, T | A, S | − | + |
| 38 | 2 | 93 | D, T | A, S | − | − |
|  |  | 94 | D, T | A, S | − | − |
| 39 | 5 | 95 | D, T | A | − | − |
|  |  | 96 | D, T | A, S | − | − |
|  |  | 97 | T | S | − | − |
|  |  | 98 | D, T | S | − | − |
|  |  | 99 | D | S | − | − |
| 40 | 2 | 100 | D | A | − | + |
|  |  | 101 | D | A | − | + |

Note: −, negative; +, positive; ND, not determine; D, *Dactylogyrus* spp.; G, *Gyrodactylus* spp.; T, *Trichodina* spp.; Ich, *Ichthyobodo* spp.; Scy, *Scyphidia* spp.; Ch, *Chilodonella* spp.; M, *Myxobolus* spp.; A, *Aeromonas* spp.; S, *Streptococcus* spp.; Fl, *Flavobacterium* spp.; Fr, *Francisella* spp.; TiLV, Tilapia lake virus; TiPV, Tilapia parvo virus.
