# Supplementary material for: Distribution and Risk Factors Associated With Tilapia Parvovirus (TiPV) Presence in Red Hybrid Tilapia (Oreochromis spp.) Farms in Thailand
Source: Transbound Emerg Dis. 2025 Feb 10;2025:6618755. doi: 10.1155/tbed/6618755 (PMC12016865; doi:10.1155/tbed/6618755)
Supplement: Supporting Information 2 — Questionnaire in English and Thai. [file 6618755.f2.docx]

**Questionnaire**

**Part 1 Farm information**

**Date__________________**

**Farm’s name** ______________**Owner’s name** ____________________**Phone number**______________________

**Contact person** __________________**Phone number**_________________________________________________

**Location of farm**_______________Subdistrict______________District_______________Province_____________

**Culture system**

❑ **Floating cages in the river** Source of water ______________________

Frequency of nylon net cleaning during the production cycle ______________________

Method of cleaning the cage after the production cycle

❑ No cleaning ❑ High-pressure water cleaning ❑ Sun drying

❑ High-pressure water cleaning and sun drying

❑ High-pressure water cleaning, soaking in a disinfectant and sun drying

❑ **Floating cages in the earthen pond** Source of water ______________________

Frequency of nylon net cleaning during the production cycle ______________________

The frequency of water changes ______________________

Method of cleaning the cage after the production cycle

❑ No cleaning ❑ High-pressure water cleaning ❑ Sun drying

❑ High-pressure water cleaning and sun drying

❑ High-pressure water cleaning, soaking in a disinfectant and sun drying

❑ **Earthen pond** Source of water ____________________________________________

The frequency of water changes _______________________________

❑ **Concrete pond** Source of water ____________________________________________

The frequency of water changes _______________________________

❑ **Othe**r (Please specify) ____________Source of water ___________________________________________

The frequency of water changes _______________________________

**The total number of ponds/floating cages within the farm____________________________________**

**Is red hybrid tilapia the sole species in the containment?** ❑ Yes ❑ No (Please specify) __________

**Are other aquatic animal species raised on the farm?** ❑ Yes ❑ No (Please specify) ___________

**Disposal of dead fish** (1-2 methods are most used)

❑ Do not remove carcasses ❑ Remove carcasses for other aquatics consume

❑ Discarding into public water ❑ Remove carcasses for burning, fermenting, or burial

**Does the farm use aerators?** ❑ Yes ❑ No

**Aerator operation** ❑ Used daily ❑ Used only when fish show signs of illness

**Type of fish feed** ❑ Commercial pellets ❑ Other (Please specify) ______________

**Feeding control method** ❑ % feed intake relative to fish body weight ❑ Ad libitum

**Pond preparation before use**_____________________________________________________________________

**Water treatment before use**______________________________________________________________________

**Part 2** **Information about the sampled fish**

**Lot/zone**___________________

**Source of hatchery** _________________________ **The date of receiving fish into the farm** ______________

**The weight of the fish before culture**_____________ g **Day of culture**______________ day

**The number of fish/cages; pond** ________________fish **Size of cage; pond** (W×L×D)________________m **Depth of water**________________ m

**The date when the fish began to show clinical signs or death**________________ **Mortality rate** _____________

**Clinical signs**__________________________________________________________________________________

__________________________________________________________________________________________________________________________________________________________________________________________

__________________________________________________________________________________________________________________________________________________________________________________________

**ส่วนที่ 1 ข้อมูลฟาร์ม**

**แบบสอบถาม**

วันที่__________________

**ชื่อฟาร์ม**____________________**ชื่อเจ้าของฟาร์ม**_______________________________**เบอร์โทรติดต่อ**____________________________

**สัตวแพทย์/นักวิชาการประจำฟาร์ม** _____________________________**เบอร์โทรติดต่อ**__________________________________________

**ที่ตั้งฟาร์ม**____________________________ตำบล_________________________อำเภอ__________________จังหวัด_________________

**ลักณะการเลี้ยง**

❑ กระชังในแม่น้ำ แหล่งน้ำที่ใช้เลี้ยง ______________________

ความถี่ในการทำความสะอาดมุ้งฟ้าระหว่างการเลี้ยง _______________________

การทำความสะอาดกระชังระหว่างรุ่นการเลี้ยง ❑ ไม่ได้ทำ ❑ ฉีดล้าง ❑ ตากแดด ❑ ฉีดล้างและตากแดด

❑ ฉีดล้าง แช่น้ำยาฆ่าเชื้อ และตากแดด

❑ กระชังในบ่อดิน แหล่งน้ำที่ใช้เลี้ยง___________________

ความถี่ในการทำความสะอาดมุ้งฟ้า_____________________ ความถี่ในการถ่ายน้ำ _____________________

การทำความสะอาดกระชังระหว่างรุ่นการเลี้ยง ❑ ไม่ได้ทำ ❑ ฉีดล้าง ❑ ตากแดด ❑ ฉีดล้างและตากแดด

❑ ฉีดล้าง แช่น้ำยาฆ่าเชื้อ และตากแดด

❑ บ่อดิน แหล่งน้ำที่ใช้เลี้ยง______________________ ความถี่ในการถ่ายน้ำ _____________________

❑ บ่อคอนกรีต แหล่งน้ำที่ใช้เลี้ยง_______________________ ความถี่ในการถ่ายน้ำ _____________________

❑ อื่นๆ (ระบุ)_____________ แหล่งน้ำที่ใช้เลี้ยง_______________________ ความถี่ในการถ่ายน้ำ _____________________

**จำนวน กระชัง;บ่อ ทั้งหมดภายในฟาร์ม**________________________________________________________________________________________

**ชนิดของปลาที่เลี้ยง**

ในกระชัง/บ่อ มีเฉพาะปลานิลเท่านั้นหรือไม่ ❑ ใช่ ❑ ไม่ใช่ (ระบุ) __________________

ในฟาร์มมีการเลี้ยงสัตว์น้ำชนิดอื่นหรือไม่ ❑ ใช่ ❑ ไม่ใช่ (ระบุ) __________________

**วิถีการกำจัดซากปลาตาย** (1-2 วิธีที่ทำบ่อยที่สุด)

❑ ไม่เก็บออก ❑ ตักออกให้ปลาอื่นกิน ❑ ตักออกทิ้งนอกกระชัง/บ่อ ❑ ตักออกไปทำลาย (เผา; ฝัง; หมัก)

**เครื่องให้อากาศ**  ❑ มี ❑ ไม่มี

**การเปิดเครื่องให้อากาศ** ❑ เปิดเป็นประจำทุกวัน ❑ เปิดเฉพาะช่วงปลามีอาการขาดออกซิเจน

**ประเภทอาหาร** ❑ อาหารสำเร็จรูป ❑ อื่นๆ (ระบุ) _____________________

**วิธีการควบคุมการให้อาหาร**  ❑ คำนวณจากน้ำหนักตัวของปลา ❑ ให้ตามการกินได้ของปลา

**วิธีการเตรียมบ่อก่อนเลี้ยง**___________________________________________________________________________________________

**วิธีการเตรียมน้ำก่อนเลี้ยง**___________________________________________________________________________________________

**ส่วนที่ 2 ข้อมูลสัตว์ป่วย**

**รุ่น/โซน___________________**

**แหล่งที่มาลูกพันธุ์**_________________________ **วันที่รับปลาเข้าฟาร์ม**______________

**ขนาดของปลาที่รับเข้าฟาร์ม**__________________กรัม **ลงปลามาแล้วกี่วัน**______________วัน

**จำนวนปลา**/กระชัง;บ่อ ____________ตัว **ขนาด** (กว้าง×ยาว×ลึก)_____________________เมตร;ไร่

**ความลึกของน้ำ**________________เมตร

**วันที่เริ่มป่วย/ตาย**____________________________**จำนวนป่วยตาย**____________________________________

**อาการป่วยโดยละเอียด**____________________________________________________________________________________________

______________________________________________________________________________________________________________

______________________________________________________________________________________________________________
